# Characterization of fungal microbial diversity in healthy and diarrheal Tibetan piglets

**DOI:** 10.1186/s12866-021-02242-x

**Published:** 2021-07-03

**Authors:** Qinghui Kong, Suozhu Liu, Aoyun Li, Yaping Wang, Lihong Zhang, Mudassar Iqbal, Tariq Jamil, Zhenda Shang, Lang-sizhu Suo, Jiakui Li

**Affiliations:** 1 College of Veterinary Medicine, Huazhong Agricultural University, 430070 Wuhan, People’s Republic of China; 2 College of Animal Science, Tibet Agricultural and Animal Husbandry University, 860000 Nyingchi, People’s Republic of China; 3 Tibetan Plateau Feed Processing Engineering Research Center, 860000 Nyingchi, People’s Republic of China; 4 Faculty of Veterinary and Animal Sciences, The Islamia University of Bahawalpur, 63100 Bahawalpur, Pakistan; 5 Institute of Bacterial Infections and Zoonoses, Friedrich-Loeffler-Institut, 07743 Jena, Germany

**Keywords:** Microbial diversity, Fungus, ITS, Diarrhea, Tibetan pig

## Abstract

**Background:**

Diarrhea is an important ailment limiting the production of the Tibetan pig industry. Dynamic balance of the intestinal microbiota is important for the physiology of the animal. The objective of this work was to study fungal diversity in the feces of early weaning Tibetan piglets in different health conditions.

**Results:**

In the present study, we performed high-throughput sequencing to characterize the fungal microbial diversity in healthy, diarrheal and treated Tibetan piglets at the Tibet Autonomous Region of the People’s Republic of China. The four alpha diversity indices (Chao1, ACE, Shannon and Simpson) revealed no significant differences in the richness across the different groups (*P* > 0.05).

In all samples, the predominant fungal phyla were Ascomycota, Basidiomycota and Rozellomycota. Moreover, the healthy piglets showed a higher abundance of Ascomycota than the treated ones with a decreased level of Basidiomycota. One phylum (Rozellomycota) showed higher abundance in the diarrheal piglets than in the treated. At genus level, compared with that to the healthy group, the proportion of *Derxomyces* and *Lecanicillium* decreased, whereas that of *Cortinarius* and *Kazachstania* increased in the diarrheal group. The relative abundances of *Derxomyces*, *Phyllozyma* and *Hydnum* were higher in treated piglets than in the diarrheal ones.

**Conclusions:**

A decreased relative abundance of beneficial fungi (e.g. *Derxomyces* and *Lecanicillium*) may cause diarrhea in the early-weaned Tibetan piglets. Addition of probiotics into the feed may prevent diarrhea at this stage. This study presented the fungal diversity in healthy, diarrheal and treated early-weaned Tibetan piglets.

**Supplementary Information:**

The online version contains supplementary material available at 10.1186/s12866-021-02242-x.

## Background

Tibetan pig is an indigenous swine breed of the Tibet Autonomous Region and adjacent areas (e.g. Sichuan, Gansu and Yunnan provinces) in People’s Republic of China (PRC) at approximately 3000 m above sea levels. It is among the pig breeds that adapted to the local cold and high-altitude environment[1, 2]. These pigs have become herbivorous, making them indispensable for pig production at the plateau [3]. Diarrhea is a major health problem in piglets worldwide posing economic threat due to its negative impact on growth and reproductive parameters and mortality [4–6]. It may be of infectious (e.g. bacteria, viruses, etc.) and/or non-infectious origin (e.g. intestinal dysfunction) in early-weaned piglets [7]. Intestines are one of the major organs for feed digestion and absorption, where gut microbiota plays an important role in maintaining the physiology [8]. This microbiota is a complex, dense and actively metabolic microbial community system [9] which provides balance to the host nutrition, metabolism and intestinal immunity [10, 11]. It is mainly composed of bacteria including some viruses, protozoa and fungi. The later are unicellular or multicellular heterotrophs that absorb nutrients in a parasitic, saprophytic or symbiotic manner resulting in a beneficial, pathogenic or opportunistic relationship with the host [12]. Parasitic fungal diseases are of major scientific interest especially in immunocompromised patients, those treated with long-term antimicrobial therapy and the populations living under humid climate. Therefore, analyzing and characterizing the fungal community structure becomes crucial regarding animal health especially at poor herdsman's level. Tibetan pigs provide an important economic and social benefits to the nomadic Tibetan population.

Given to the harsh climate and extensive farming practices, the piglets often suffer postweaning diarrhea, which severely reduces the production performance and economic benefits to the local farmers. Owing to the scarce literature on the gut mycobiome in weaning piglets, we were interested to know the intestinal fungal diversity in healthy, diarrheal and treated Tibetan piglets in Nyingchi, Tibet Autonomous Region of the PRC to provide a theoretical reference for the prevention and treatment regimen  in these piglets.

## Methods

### Ethics statement

This study was approved and instructed by the Tibetan Pig Collaborative Research Center of Tibet Agricultural and Animal Husbandry University, Tibet, China (unified social credit code: 12540000MB0P013721).

### Animal feeding and sample collection

The experimental animals for this study were taken from five healthy sows of Tibetan pig breed origin (The sows were raised at Tibetan Pig Collaborative Research Center at Tibet Agriculture and Animal Husbandry University) maintained under similar conditions. The sows gave birth on the same day, and the piglets and sows reared together in a farrowing house (the temperature of the farrowing house was approximately 21℃, and the farrowing bed was cleaned and disinfected). At three weeks of age, the piglets were offered piglet feed (Supplementary Table 1). At 6 weeks of age, healthy piglets were weaned and transferred to a nursery house (the temperature of nursery house was 16℃) where they continued the piglet feed.

Hermann-Bank test criteria [13] were used to identify healthy and diarrheal piglets. Feces from healthy piglets (i.e no apparent signs of diarrhea and secreting granular stripe-shaped feces for more than two days) and diarrheal piglets (i.e. secreting thin and unformed feces for more than two days) were collected, marked and transferred from the ranch to the laboratory using a vehicle-mounted refrigerator (-15℃). The samples were stored at -20℃ until further analysis. The marked diarrheal piglets were treated by injection into the neck muscle with 1 mL of (4%) gentamycin sulfate (Shijiazhuang Huaxu Pharmaceutical Co. Ltd., Shijiazhuang, PRC; product number: 17,925,752,842). Five fecal samples each from healthy (group A; marked as A1, A2, A3, A4, and A5), treated (group B; marked as B1, B2, B3, B4, and B5), and diarrheal piglets (group C; marked as C1, C2, C3, C4, and C5) were selected and processed for further analysis.

### DNA extraction

Genomic DNA was extracted from the fecal samples by using QIAamp Fast DNA Stool Mini Kit (Qiagen, Hilden, Germany) according to the manufacturer’s recommendations. The concentration and quality of the DNA were determined by NanoDrop 2000 (Thermo Fisher Scientific Inc., Massachusetts, USA) and 1.2 % agarose gel electrophoresis, respectively.

### Internal transcribed spacer (ITS) hypervariable region gene amplification

Specific polymerase chain reaction (PCR) primers (ITS5F: GGAAGTAAAAGTCGTAACAAGG and ITS2R: GCTGCGTTCTTCATCGATGC) with special barcodes on the forward primer were used. The total genomic DNA was uniformly diluted to 20 ng/µL and used as a template. The PCR protocol was performed with 30 cycles of 98℃ (15 s), 55℃ (30 s), 72℃ (30 s), and 72℃ (5 min). The PCR amplification products were detected via 2 % agarose gel electrophoresis, and the target fragments were recovered using the AxyPrep DNA Gel Extraction Kit (Axygen Inc., Union City, CA, USA). The recovered PCR products were detected by the Quant-iT PicoGreen dsDNA Assay Kit (Invitrogen Co., Carlsbad, CA, USA).

### Library preparation and sequencing

The sequencing library was constructed using the TruSeq Nano DNA LT Library Prep Kit (Illumina, Inc., San Diego, CA, USA). The sequencing library was selected and purified GeneJET Gel Extraction Kit (Thermo Fisher Scientific, Waltham, MA, USA) by 2 % agarose gel electrophoresis, and the quality was tested by the Agilent High-Sensitivity DNA Kit (Agilent Technologies, Inc., Santa Clara, CA, USA). Then, paired-end sequencing of the qualified sequencing library was performed using Illumina MiSeq equipment (MiSeq Reagent Kit V3; Personalbio, Shanghai, China).

### Sequence data processing and statistical analysis

Sequence analysis data were established as operational taxonomic units (OTUs) via Uclust with a 97 % similarity [14], and the most abundant sequence in each OTU was selected as the representative sequence [15]. Then, OTUs were taxonomically classified and grouped by comparison with those in the Unite database [16]. The richness and evenness index of microbial flora were calculated using the measurement indices (Chao1, ACE, Shannon, and Simpson); beta diversity based on the weighted UniFrac distance matrices was calculated with QIIME (Version 1.7.0), and cluster analysis was preceded by principal coordinate analysis (PCA) [17]. The Metastats statistical algorithm was used to analyze the discrepancy in microbial communities between groups at the phylum and genus levels [18]. The heat map was created via R software (v3.0.3). The data were evaluated statistically by one-way analysis of variance.

## Results

### Sequencing results and operational taxonomic unit (OTU) cluster statistical analysis

**Fig. 1:**
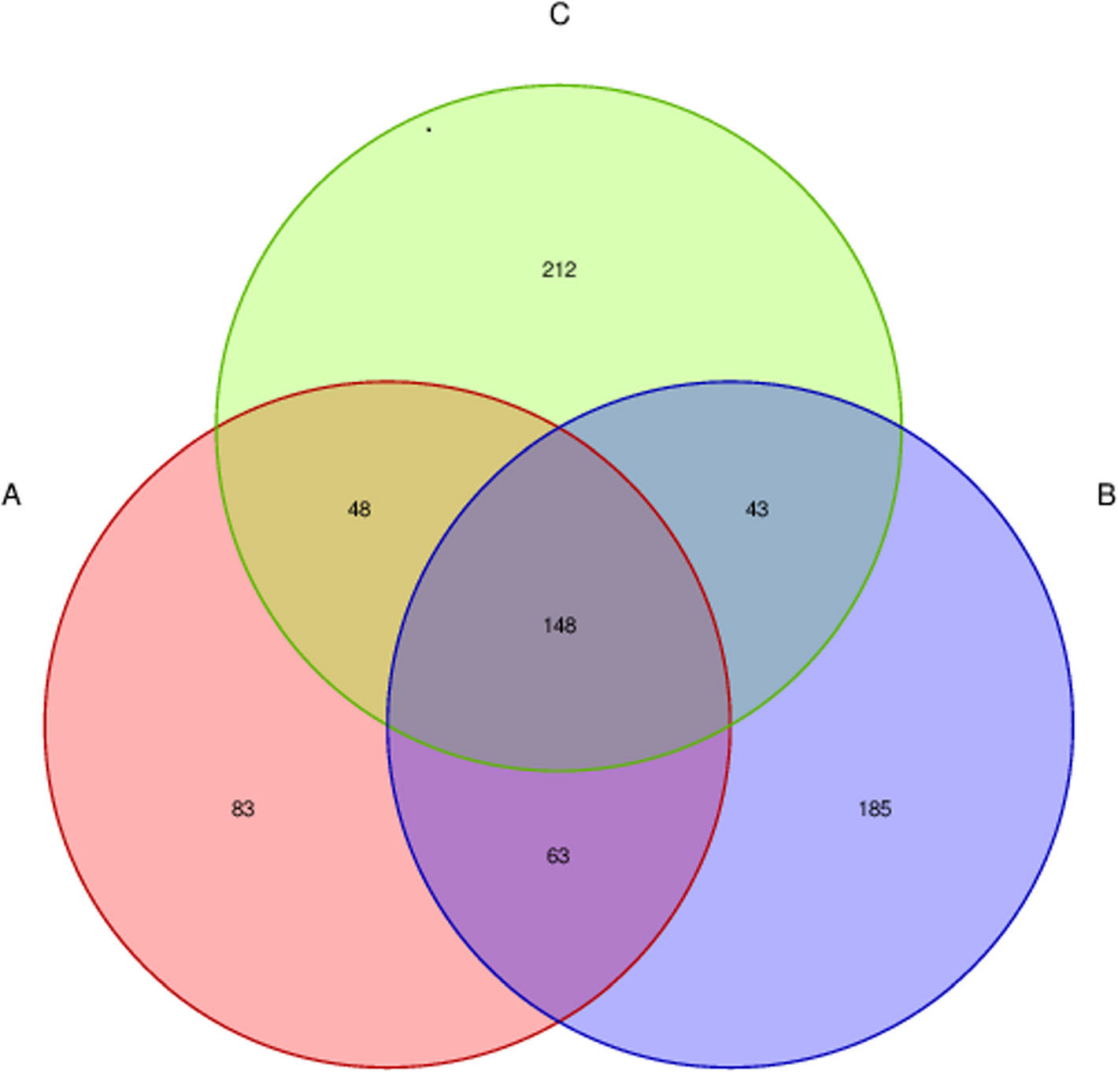
Venn diagram analysis of the fecal microflora of weaning piglets of different groups. A: healthy piglets; B: treated piglets; C: diarrheal piglets

In the present study, a total of 1,297,417 high-quality sequences were obtained from 15 fecal samples, and the average effective combined sequence was 86,494 for each sample (Supplementary Figure S1). The distribution length in each sample was 100–400 bp (Supplementary Figure S1). The sequences were established at the phylum, class, order, family, genus and species levels as OTUs via Uclust with over 97 % similarity (Supplementary Figure S1). At the species level, ≥ 200 OTUs were identified. The three groups shared 148 fungal species, as found by Venn map/diagram analysis (Fig. 1). The diarrheal piglets showed 212 common fungal species, which were not found in the healthy and antimicrobial-treated piglets. A total of 83 fungal species were found to be common among the healthy piglets.

### Microbial community diversity of Tibetan piglets in different groups

**Fig. 2:**
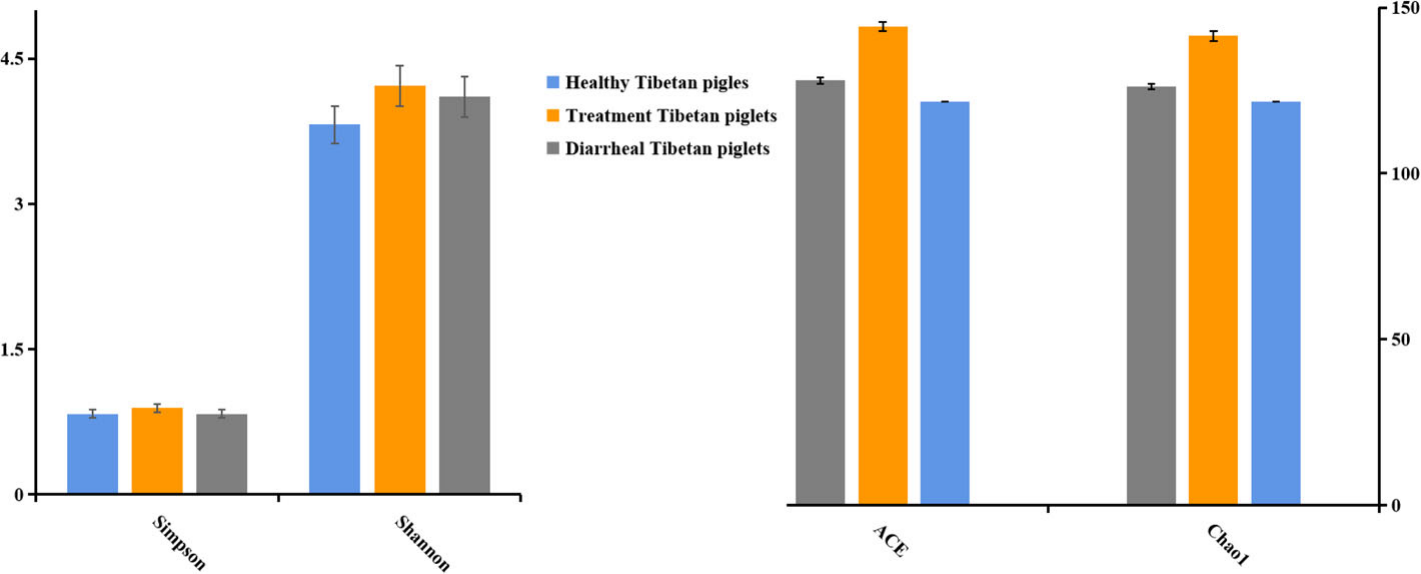
Diversity indices of the fecal microbiota in different Tibetan piglets. Chao1, ACE, Shannon, and Simpson indices were used to evaluate the alpha diversity of the fecal microbiota

**Fig. 3:**
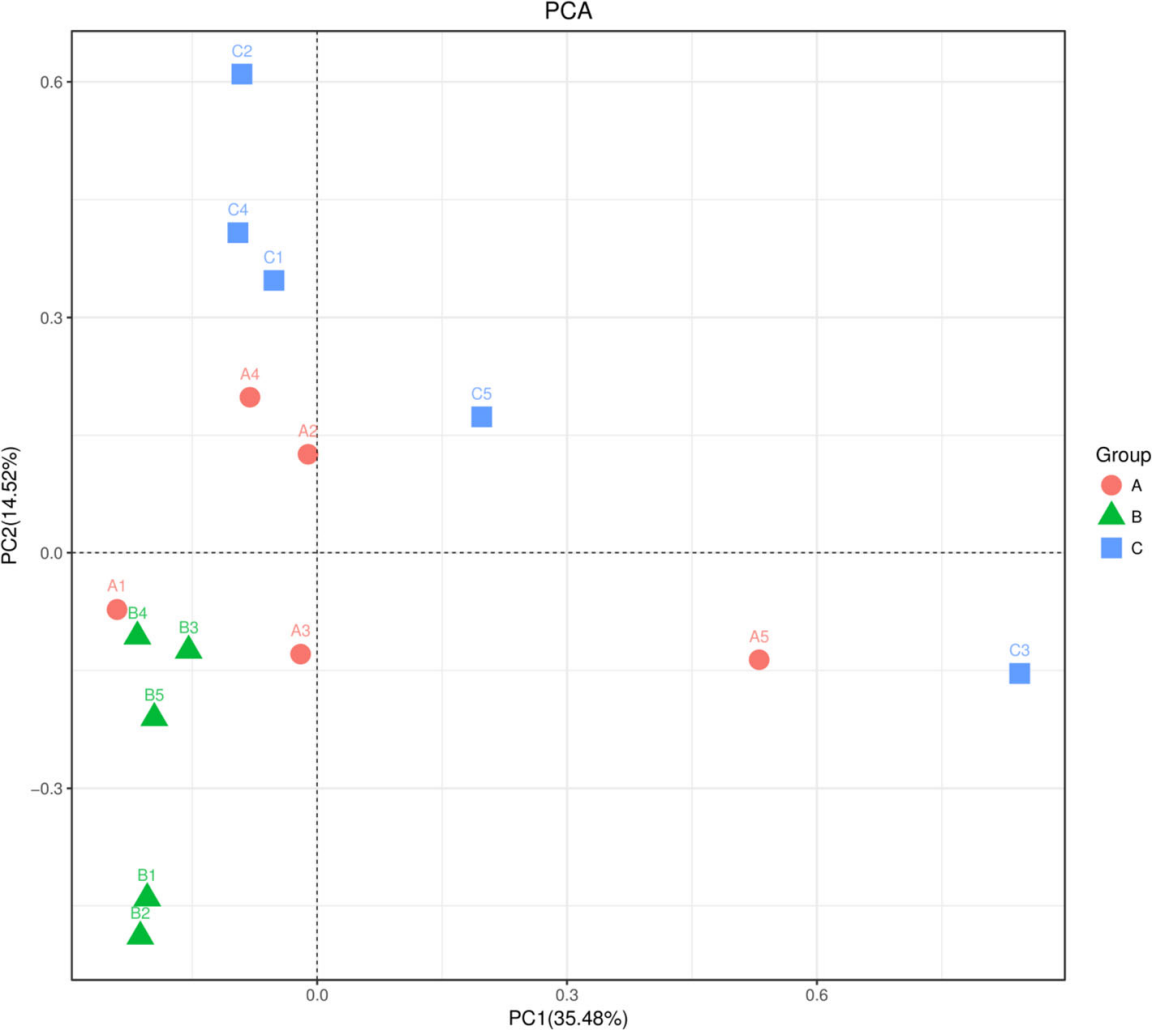
Principal component analysis of the fecal microbiota. PCA map based on Euclidean distance. Each point indicates one sample. The distance of the two points indicates the difference of fecal microbiota. A: healthy piglets; B: treated piglets; C: diarrheal piglets

The requirements for sequencing and analysis were met by confirming the sequence numbers by the presence line in the rank abundance curve, evenness of the microbial species and the plateau phase of the Chao1 and Shannon curves (Supplementary Figure S2; A, B). The Simpson index reached 0.85, 0.88 and 0.83 in the healthy piglet, treated piglet and diarrheal piglet groups, respectively. The Simpson index in the healthy piglet group was lower than that in the treated piglet group, whereas the Simpson index in the treated piglet group was higher than that in the diarrheal piglet group. However, no significant difference was observed among the three groups (*P* > 0.05) (Fig. 2). The Shannon indices of the three groups were 3.81 (A), 4.21 (B) and 4.10 (C), with no significant difference among them. The Simpson and Shannon indices demonstrated that there was no obvious difference among all the samples (Fig. 2). The Chao1 and ACE indices were 121.79 and 123.97, 141.39 and 144.26, and 126.09 and 127.99 for groups A, B and C, respectively. However, no significant difference was observed in the two indices among the different groups (*P* > 0.05) (Fig. 2). The Chao1 and ACE indices revealed no striking difference in fungal microbial evenness among the different groups (Fig. 2). However, significant differences were found in the fungal structure by principal component analysis (PCA) in different groups, especially among diarrheal piglets and treated piglets (Fig. 3).

### Microbial community structure of Tibetan piglets in different groups

**Fig. 4:**
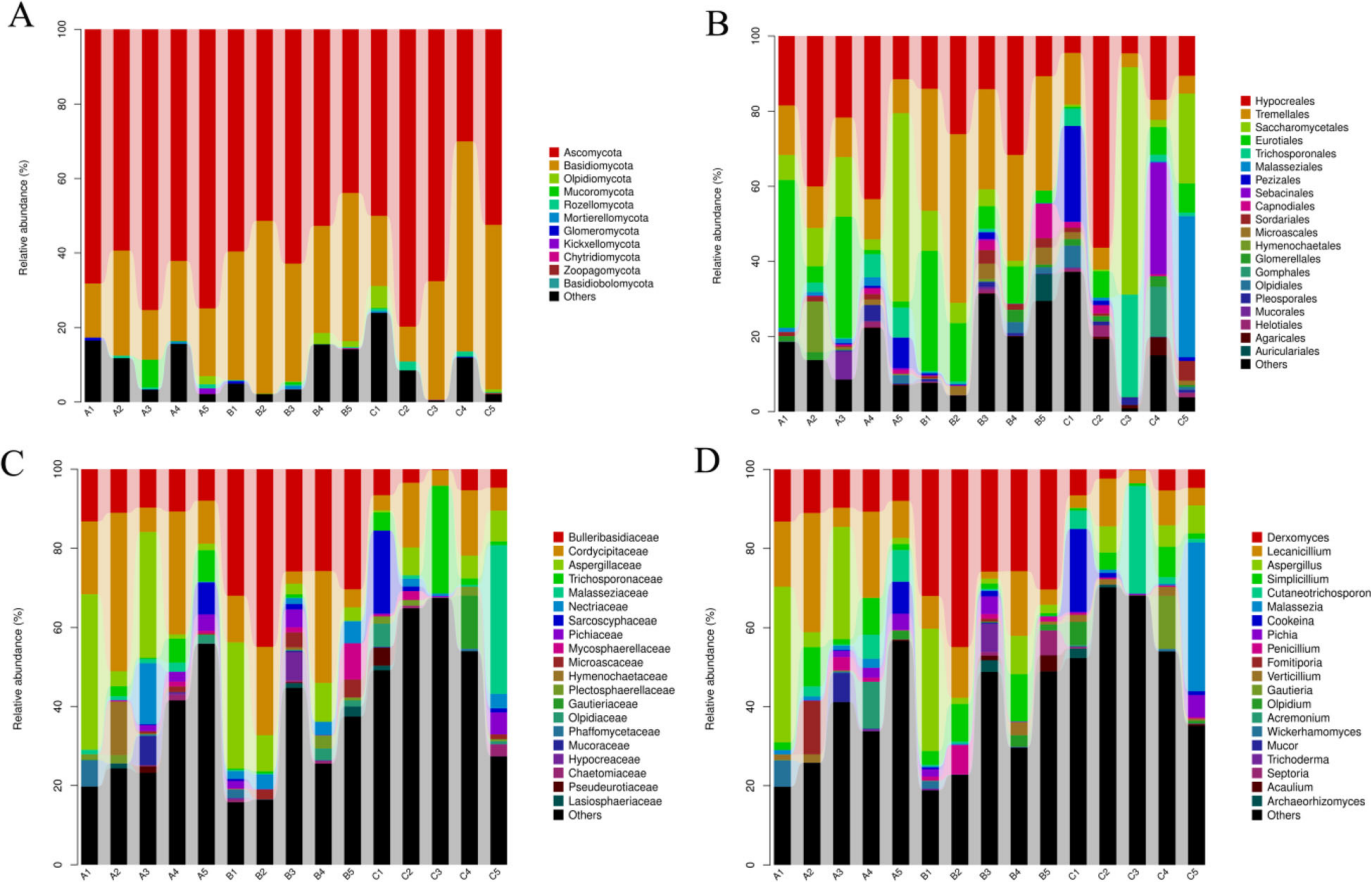
Microbial community structure at the phylum level (**A**), order level (**B**), family level (**C**), and genus level (**D**) in different Tibetan piglets. A1-A5: healthy piglets; B1-B5: treated piglets; C1-C5: diarrheal piglets

The microbial community structure according to the classification hierarchy is shown in Supplementary Figure S3. Ascomycota (67.96 % in group A, 54.10 % in group B, 55.94 % in group C) and Basidiomycota (19.10 % in group A, 36.18 % in group B, 32.18 % in group C) showed dominance in microbiota composition at the phylum level in all piglets (Fig. 4 A). At the order level, *Hypocreales* was found to be more abundant in healthy piglets (26.98 %) than in treated and diarrheal piglets (19.34 % in group B and 18.60 % in groups C), *Tremellales* was found to be more abundant in treated piglets (32.50 %) than in healthy piglets and diarrheal piglets (10.36 % in group A and 6.64 % in group C), and *Saccharomycetales* was found to be less abundant in treated piglets (4.42 %) than in healthy piglets and diarrheal piglets (17.14 % in group A and 17.44 % in group C) (Fig. 4B). At the family level, *Bulleribasidiaceae* was found to be more abundant in treated piglets (31.80 %) than in healthy piglets and diarrheal piglets (9.92 % in group A and 4.04 % in group C), while *Cordycipitaceae* was found to be more abundant in healthy piglets (21.4 %) than in treatment piglets and diarrheal piglets (14.02 % in group B and 9.28 % in group C) and *Aspergillaceae* was found to be less abundant in diarrheal piglets (4.24 %) than in healthy piglets and treatment piglets (15.52 % in group A and 11.38 % in group B) (Fig. 4 C). At the genus level, *Derxomyces* was more abundant in group B (31.42 %) than in group A (9.92 %) and group C, while *Lecanicillium* and *Aspergillus* were more abundant in healthy piglets (16.14 and 13.96 %, respectively) than in diarrheal piglets and treatment piglets (8.6 % in group B and 5.36 % in group C; 9.52 % in group B and 3.86 % in group C, respectively) (Fig. 4D).

### Comparison of the microbial diversity in each piglet group

**Fig. 5:**
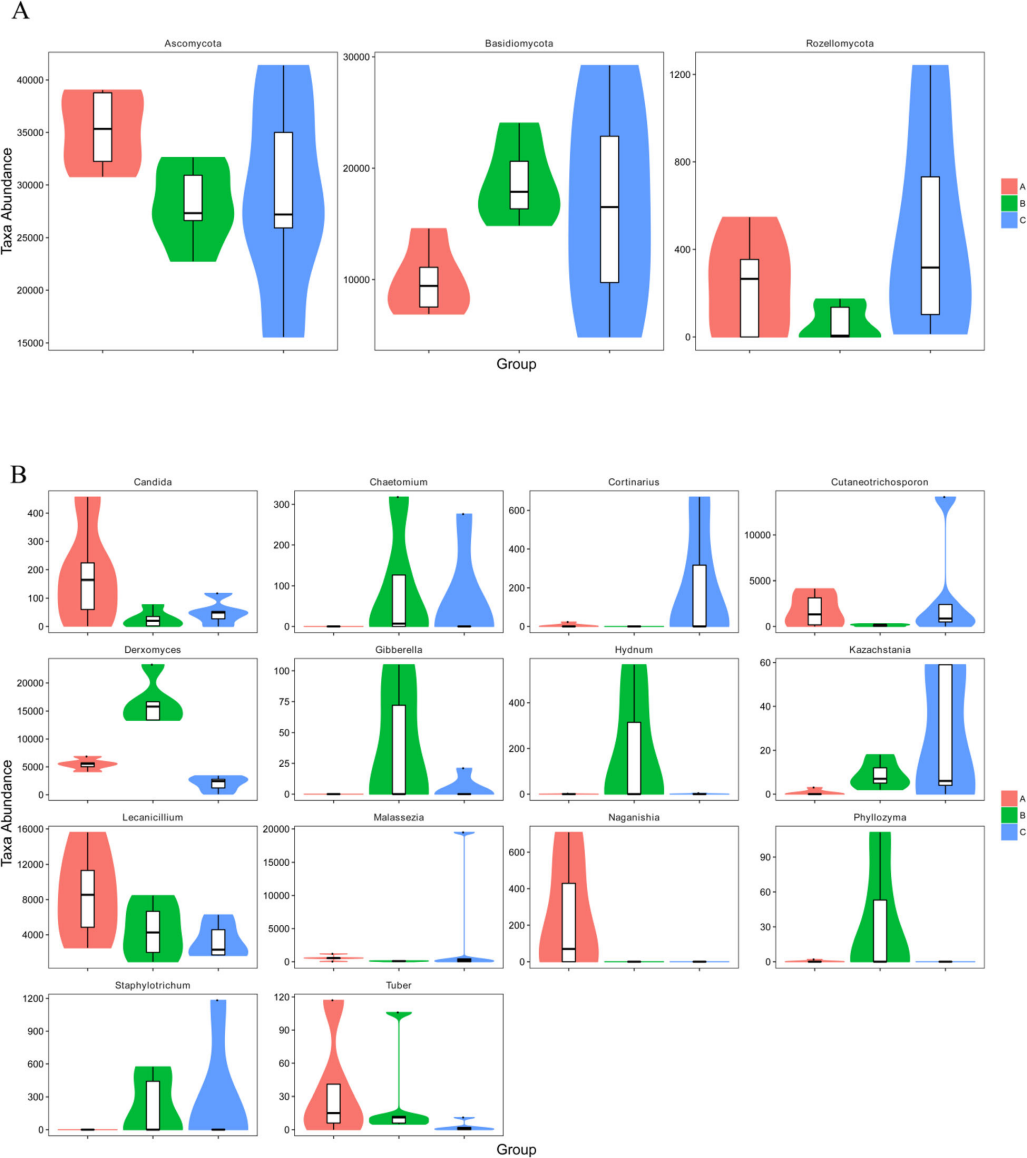
Composition of microbial diversity at the phylum level (**A**) and genus level (**B**) in each piglet group, as determined by Metastats. A: healthy piglets; B: treated piglets; C: diarrheal piglets

**Fig. 6:**
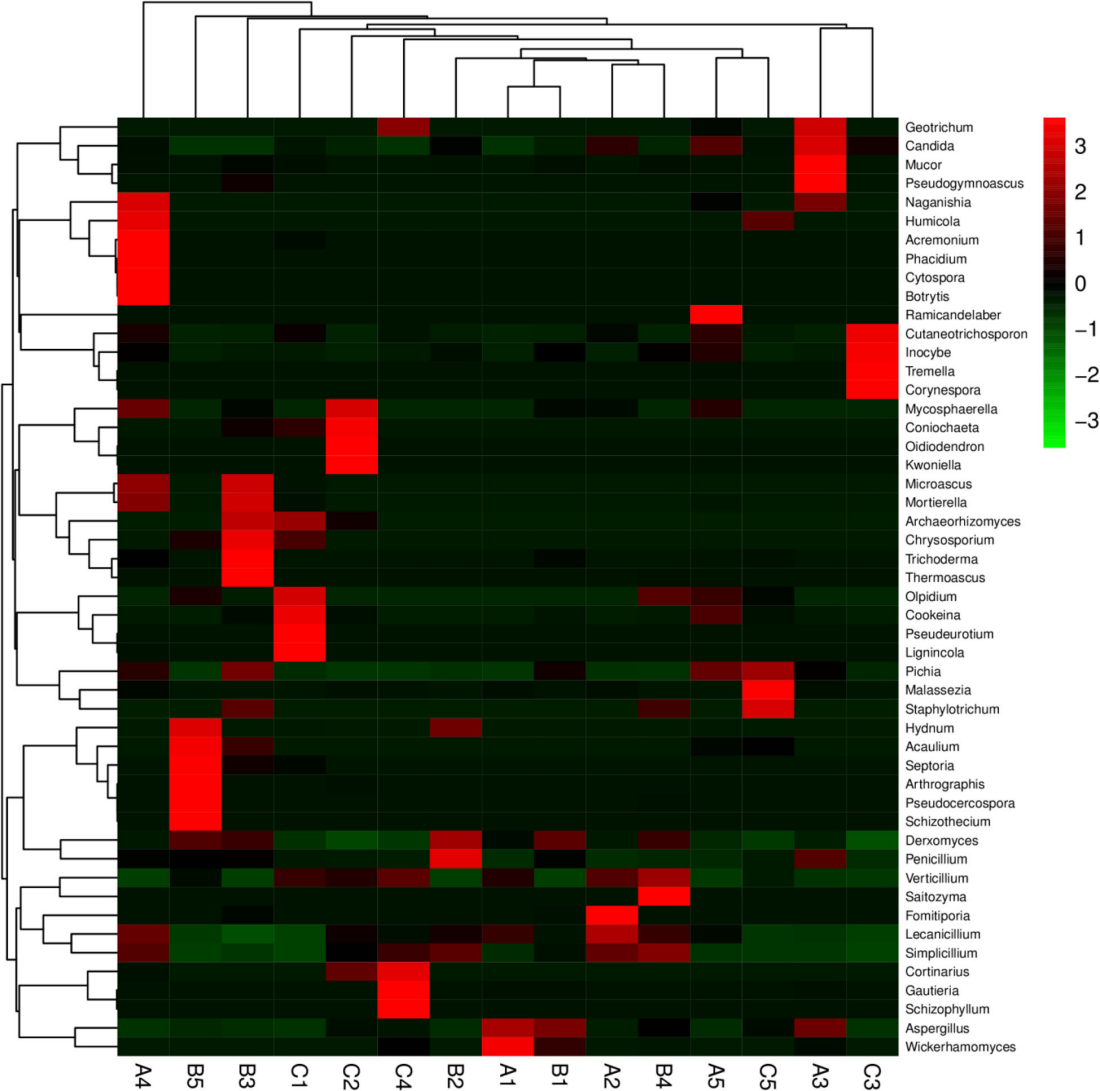
Heat map of the fifty most abundant genera in each Tibetan piglet sample. A1-A5: healthy piglets; B1-B5: treated piglets; C1-C5: diarrheal piglets

At the phylum level, Ascomycota and Basidiomycota were significantly different between groups A and B (*P* < 0.01) (Fig. 5 A). Rozellomycota exhibited a significant difference between groups B and C (*P* < 0.05) (Fig. 5 A). At the genus level, 11 genera presented significant differences between groups A and B. Among them, *Derxomyces*, *Malassezia* and *Kazachstania* showed significant differences at *P* < 0.01, whereas *Candida*, *Naganishia*, *Lecanicillium*, *Cutaneotrichosporon*, *Staphylotrichum*, *Gibberella*, *Chaetomium* and *Phyllozyma* showed significant differences at *P* < 0.05. Similarly, six genera significantly differed between groups A and C. Among them, *Derxomyces* and *Naganishia* were significantly different at *P* < 0.01, whereas *Kazachstania*, *Tuber*, *Cortinarius* and *Lecanicillium* were significantly different at *P* < 0.05. Similarly, four genera showed significant differences between groups B and C. Among them, *Derxomyces* significantly differed at *P* < 0.01, whereas *Cortinarius*, *Phyllozyma* and *Hydnum* exhibited a significant difference at *P* < 0.05 (Fig. 5B). The heat map analysis showed a significant difference in *Lecanicillium* and *Derxomyces* among the three groups (Fig. 6).

## Discussion

Animal intestinal microbiota is known to influence animal health and physiology. The intestinal epithelial mucosa plays role in maintaining animal mucosal immunity [14, 19]. Therefore, changes in the diversity of the intestinal microbiota will affect intestinal function, animal health and may cause disease. Moreover, prolonged use of antimicrobials and immunosuppressants provide opportunities for the growth and reproduction of pathogenic fungi [20, 21]. As Tibetan piglets are important source of economic livelihood for the local people, studying diarrhea-causing pathogens in these piglets was meaningful.

In the current study, a variety of fecal fungi were found in the Tibetan piglets. By Venn diagram analysis, 212 fungal species were found to be shared among the diarrheal piglets, which were not found in the healthy and treated piglet groups, whereas only 83 fungal species were found in the healthy group. Significant differences were found in fungal community structure in the three groups by PCA, especially between the diarrheal and treated piglet groups. Ascomycota and Basidiomycota were the predominant phyla in weaned groups. These results were consistent with the previous studies in cattle [22], sheep [23], goats [24] and horses [25]. This finding may be related to the herbivorous characteristics of these piglets. Fungi are known for their ability to depolymerize complex molecular structures and are used in the degradation of lignocellulosic biomass, improvement of animal feed digestibility, biogas and bioethanol production, and various other applications [26]. Previous sequencing results have shown that Basidiomycota and Ascomycota were dominant phyla in the gastrointestinal tract of humans and animals [27]. At the phylum level, Rozellomycota, Basidiomycota and Ascomycota were not significantly different between the healthy and diarrheal piglets. However, Rozellomycota formed a lineage basal or sister to fungi, ancestor of Microsporidia. These species are pathogenic to animals and humans as they parasitize intestinal epithelial cells and cause diarrhea [28, 29]. Rozellomycota was found significantly more abundant in the diarrheal piglets than in the treated. Also the abundance did not differ significantly from that in the healthy piglets, indicating that it may not be associated with diarrhea in these piglets.

At the genus level, *Derxomyces*, *Lecanicillium*, *Tuber* and *Naganishia* showed significantly lower whereas *Kazachstania* and *Cortinarius* showed higher abundance in diarrheal piglets when compared to the healthy. *Derxomyces* are unicellular basidiomycete fungi that primarily reproduce through asexual reproduction by budding. Studies have shown that *Derxomyces* play an important role in agricultural production and environmental protection, produce carotenoids and astaxanthin [30] and regulated intestinal immune homeostasis in antibiotic-treated mice with diarrhea [31]. *Lecanicillium* are important biocontrol fungi used in pest control [32]. *Lecanicillium* showed higher abundance in healthy group as compared to the diarrheal and treated group. This may be due to the shift of the intestinal mycobiome towards pathogenic fungus in diarrheal piglets and the intestinal microbes was not restored to the healthy state in a timely manner after treatment. *Kazachstania* were found more abundant in diarrheal Tibetan piglets than in healthy (*P* < 0.05), which indicated that weanling stress was able to promote the growth of *Kazachstania*. *Kazachstania* are reported to produce potential peptides, formic acid and dehydroascorbic acid/vitamin C in the host intestine [33]. Under normal conditions, pigs do not require supplementation with vitamin C because they can synthesize it within themselves [34]. Nevertheless, during weaning, when animals are stressed, a lack of vitamin C may also arise in piglets [35]. Under such circumstances, the possible formation of dehydroascorbic acid by *Kazachstania* in the intestine could be beneficial for the animal. *Malassezia* was found significantly more abundant in healthy piglets (P < 0.01). This genus includes a group of opportunistic pathogenic fungi that resides on the skin of warm-blooded animals and humans. These species have a symbiotic relationship with the host and play a role in the pathogenesis of diseases such as seborrheic dermatitis, atopic dermatitis, psoriasis and pityriasis versicolor [36–38]. Further investigations are needed to confirm the relationship of this fungus in the piglet feces. *Cortinarius* is an important ectomycorrhizal genus that forms a symbiotic relationship with certain trees, shrubs and herbs [39]. Some species of *Cortinarius* have antitumor effects, while other are toxic [40–42], which shows that *Cortinarius* may cause diarrhea, as *Cortinarius* was more abundant in diarrheal piglets than in healthy and treated ones. *Tuber* was found to be significantly more abundant in healthy piglets but did not differ significantly between treated and diarrheal groups. To the best of the authors’ knowledge, information about this genus remains scarce. *Naganishia* was found more abundant in healthy piglets in our study. It is a novel fungal genus and supposedly one of the most resistant fungi prevailing in the environment [43]. Our results conveyed that the relative abundances of beneficial fungi (*Derxomyces*, *Lecanicillium*) decreased in the diarrheal piglets, which might have disrupted the normal dynamic balance of the intestinal microbiota and led to a competitive increase in the abundance of conditional pathogens (such as *Cortinarius*). This could be one of the reasons for diarrhea in weaned piglets. In addition, the abundances of *Pichia* and *Penicillium* did not change, suggesting that the two conditional pathogens were not the cause of diarrhea in weaned piglets.

To conclude, there were significant differences in gut microbial composition and structure among the three groups. A decreased relative abundance of beneficial fungi might be a factor for diarrhea in the weaning piglets. The intestinal flora was changed due to the treatment with antibiotics, and the intestinal microbes could not be restored to a healthy state in a timely manner after treatment. Alternatively, in the context of advocating non-resistant feeding, the use of probiotics would be a promising strategy to prevent and treat gastrointestinal disorders. Therefore, the addition of probiotics to the feed for weaning diarrheal Tibetan piglets would be recommended.

## Supplementary Information

**Additional file 1:**

**Additional file 2:** [44]

## Data Availability

All data generated or analyzed during this study are included in this published article. The datasets presented in this study can be found in online repositories. The names of the repository/repositories and accession number(s) can be found at: https://www.ncbi.nlm.nih.gov/, PRJNA727948.

## Abbreviations

OTU: Operational taxonomic unit
PCA: Principal component analysis
GI: Gastrointestinal
