# Supplementary material for: Characterization of fungal microbial diversity in healthy and diarrheal Tibetan piglets
Source: BMC Microbiol. 2021 Jul 3;21:204. doi: 10.1186/s12866-021-02242-x (PMC8254304; doi:10.1186/s12866-021-02242-x)
Supplement: Supplementary file 1 — Additional file 1: [file 12866_2021_2242_MOESM1_ESM.doc]

**Characterization of fungal microbial diversity in healthy and diarrheal Tibetan piglets**

Qinghui Kong^1,2,3^, Suozhu Liu^2,3^, Aoyun Li^1^, Yaping Wang^1^, Lihong Zhang^1^, Mudassar Iqbal^1,4^, Tariq Jamil^5^, Zhenda Shang^2,3^, Lang-sizhu Suo^2^, and Jiakui Li^1, 2*^

^1^College of Veterinary Medicine, Huazhong Agricultural University, Wuhan, 430070, People's Republic of China

^2^College of Animal Science, Tibet Agricultural & Animal Husbandry University, Nyingchi,860000, People's Republic of China

^3^Tibetan Plateau Feed Processing Research Center, Nyingchi, 860000, People's Republic of China

^4^Faculty of Veterinary and Animal Sciences, The Islamia University of Bahawalpur, 63100 Bahawalpur, Pakistan

^5^Institute of Bacterial Infections and Zoonoses, Friedrich-Loeffler-Institut, 07743 Jena, Germany

*Corresponding Authors: College of Veterinary Medicine, Huazhong Agricultural University, Wuhan, 430070, People's Republic of China.

E-mail addresses: 770337011@qq.com (Q. H. Kong), lijk210@sina.com (J. Li).

**
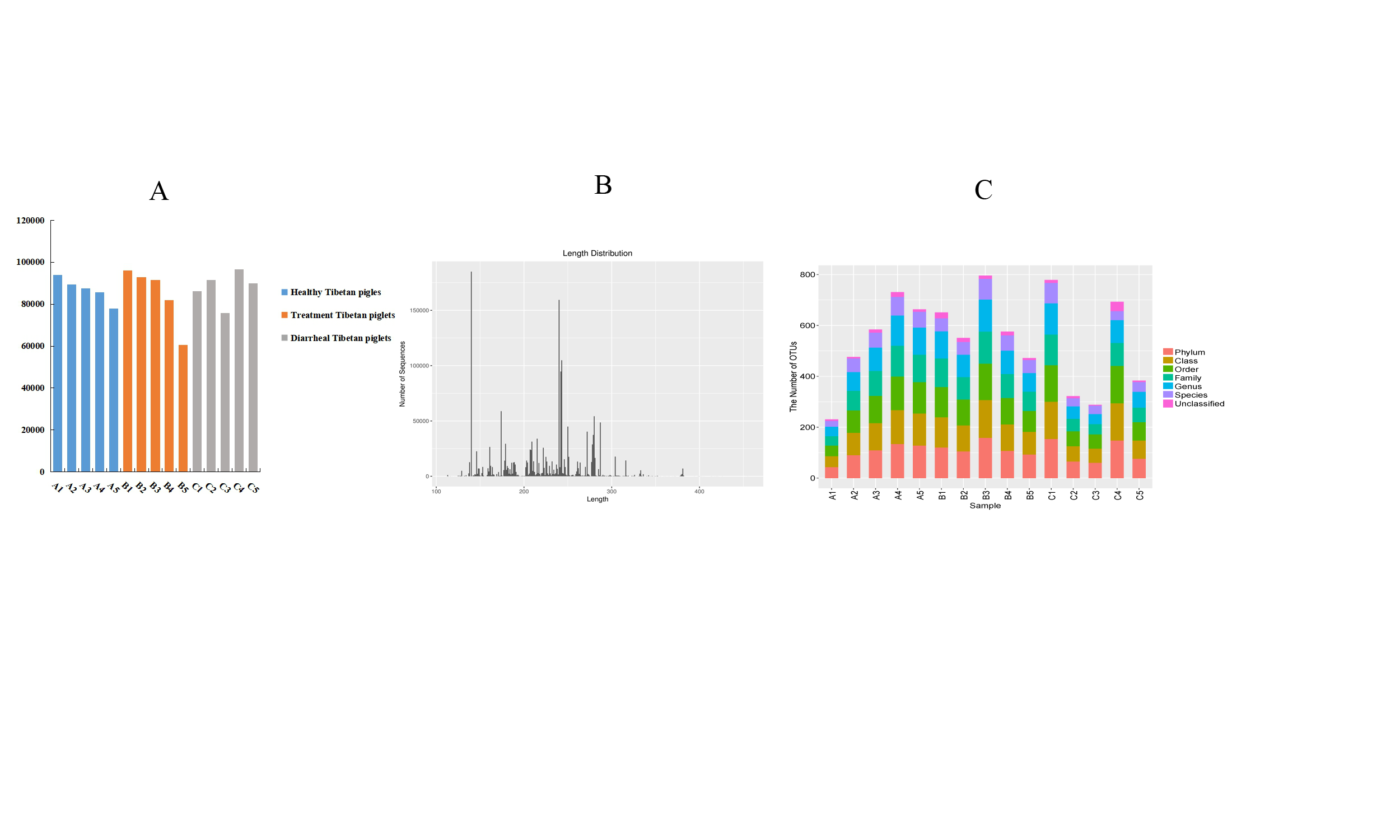
**

**Supplementary Figure S1.** Bioinformatics and statistical analysis in piglets samples. (**A**) Statistics of effective sequences. (**B**) The sequence length distribution. (**C**) Number of OTUs. **A1-A5**: Healthy piglets; **B1-B5**: Treatment piglets; **C1-C5**: Diarrheal piglets.


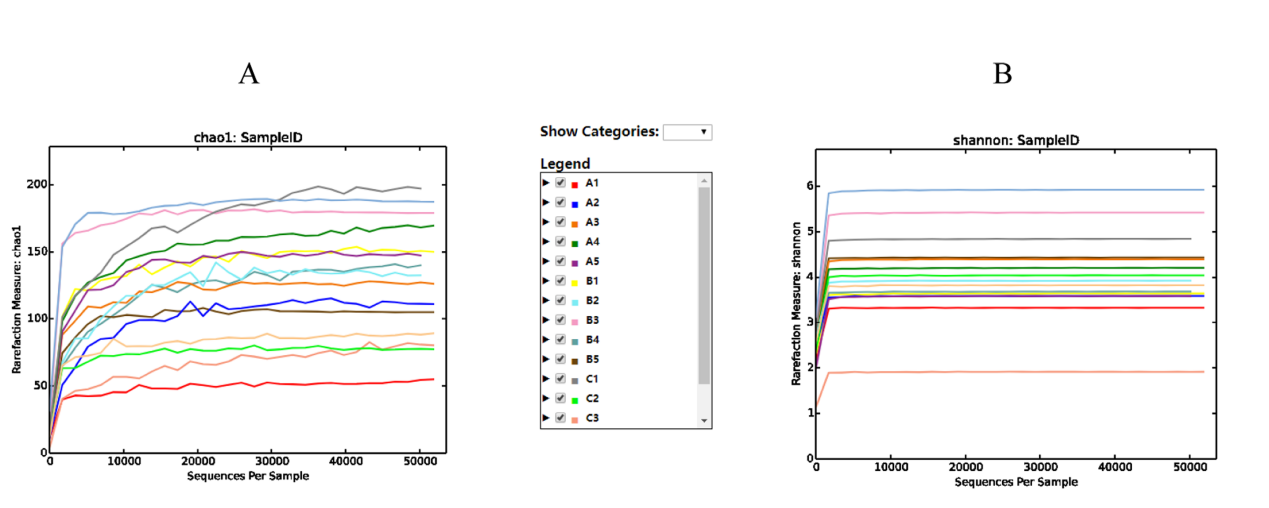


**Supplementary Figure S2.** The Microbial community diversity index curve in different piglets samples (**A**: Chao1; **B**:Shannon). **A1-A5**: Healthy piglets; **B1-B5**: Treatment piglets; **C1-C5**: Diarrheal piglets.


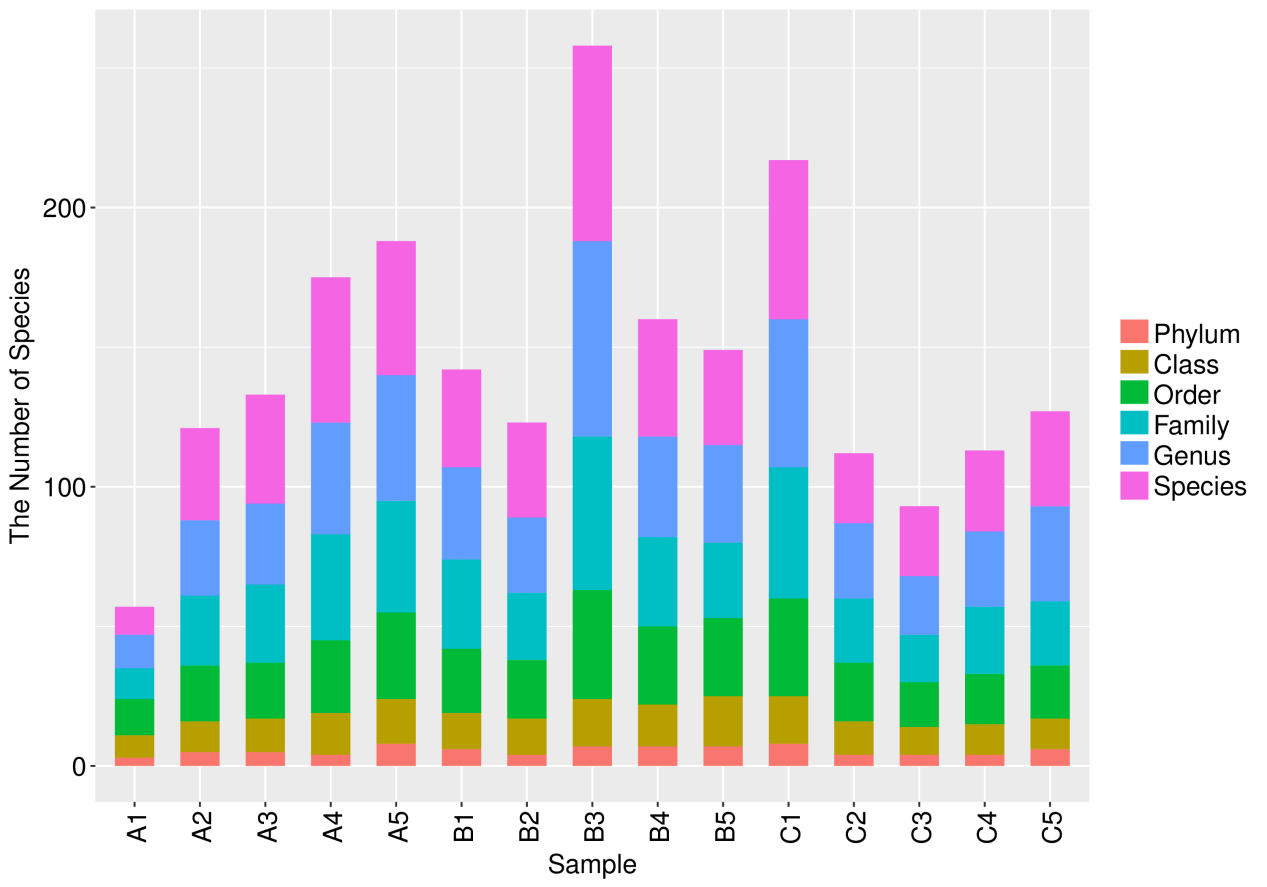


**Supplementary Figure S3.** The microbial diversity according to the classification hierarchy in each piglet group. **A1-A5**: Healthy piglets; **B1-B5**: Treatment piglets; **C1-C5**: Diarrheal piglets.
