# Supplementary material for: Characterization of fungal microbial diversity in healthy and diarrheal Tibetan piglets
Source: BMC Microbiol. 2021 Jul 3;21:204. doi: 10.1186/s12866-021-02242-x (PMC8254304; doi:10.1186/s12866-021-02242-x)
Supplement: Supplementary file 2 — Additional file 2: [44] [file 12866_2021_2242_MOESM2_ESM.doc]

**Characterization of fungal microbial diversity in healthy and diarrheal Tibetan piglets**

Qinghui Kong^1,2,3^, Suozhu Liu^2,3^, Aoyun Li^1^, Yaping Wang^1^, Lihong Zhang^1^, Mudassar Iqbal^1,4^, Tariq Jamil^5^, Zhenda Shang^2,3^, Lang-sizhu Suo^2^, and Jiakui Li^1, 2*^

^1^College of Veterinary Medicine, Huazhong Agricultural University, Wuhan, 430070, People's Republic of China

^2^College of Animal Science, Tibet Agricultural & Animal Husbandry University, Nyingchi,860000, People's Republic of China

^3^Tibetan Plateau Feed Processing Research Center, Nyingchi, 860000, People's Republic of China

^4^Faculty of Veterinary and Animal Sciences, The Islamia University of Bahawalpur, 63100 Bahawalpur, Pakistan

^5^Institute of Bacterial Infections and Zoonoses, Friedrich-Loeffler-Institut, 07743 Jena, Germany

*Corresponding Authors: College of Veterinary Medicine, Huazhong Agricultural University, Wuhan, 430070, People's Republic of China.

E-mail addresses: 770337011@qq.com (Q. H. Kong), lijk210@sina.com (J. Li).

**Supplementary Table1.** Main nutrient composition of the feed for Tibetan piglets [44].

| Product name | Crude protein  %≥ | Crude fiber  %≤ | Crude ash  %≤ | Ca% | P%≥ | NaCl% | Lys%≥ |
| --- | --- | --- | --- | --- | --- | --- | --- |
| concentrate feed | 36.0 | 8.0 | 20.0 | 1.20-3.50 | 0.90 | 0.50-2.50 | 3.20 |
